# Vibration and Sound Response of Glass-Fiber-Reinforced Polyamide 6 Using Microcellular-Foaming-Process-Applied Injection Molding Process

**DOI:** 10.3390/polym14010173

**Published:** 2022-01-02

**Authors:** Hyun Keun Kim, Jaehoo Kim, Donghwi Kim, Youngjae Ryu, Sung Woon Cha

**Affiliations:** Department of Mechanical Engineering, Yonsei University, 50, Yonsei-ro, Seodaemun-gu, Seoul 03722, Korea; sagegny@yonsei.ac.kr (H.K.K.); murja@yonsei.ac.kr (J.K.); donghwi.kim@yonsei.ac.kr (D.K.); yjryu1027@yonsei.ac.kr (Y.R.)

**Keywords:** sound and vibration, glass-fiber-reinforced plastic, microcellular foaming process, natural frequency, damping ratio

## Abstract

In this study, the vibration and sound response characteristics of composites produced via injection molding applied with a microcellular foaming process (MCPs) were improved. The study was conducted using PA6 and glass fiber composites, which are representative thermoplastic engineering plastics. Two types of specimens were used: a plate specimen to confirm the basic sound and vibration characteristics, and a large roof-rack specimen from an actual vehicle with a complex shape. The frequency response function curve was calculated by conducting an impact test, and natural frequency and damping ratio were measured based on the curve. The results confirmed that, in the case of a specimen manufactured through the injection molding process to which MCPs were applied, the natural frequency was lowered, and the damping ratio decreased. The degree of change in the natural frequency and damping ratio was confirmed. To determine the cause of the change in the natural frequency and damping ratio, the mode shape at the natural frequency of each specimen was measured and the relationship was confirmed by measuring the density and the elastic modulus of the composite. In addition, the usability of the specimens to which MCPs were applied was verified by conducting impact strength and tensile strength tests.

## 1. Introduction

To reduce the weight of products, the need for lightweight materials with advantageous physical properties is increasing. Fiber-reinforced plastics are lightweight and have various advantages, such as good physical properties, durability, rigidity, wear, and thermal properties; thus, these are increasingly being used. In particular, fiber-reinforced plastics manufactured via injection molding have good physical properties and can be produced in precise shapes; thus, they play an important role in housings and component parts, such as smartphones, electronic products, and automobiles, and their scope of application is expanding [1,2,3]. As the field of application of fiber-reinforced plastics has expanded, properties previously not required for plastics are now being required. As plastics increasingly constitute the parts of many products, particularly structures, it is necessary to study their acoustic and vibration characteristics, as well as the existing basic mechanical properties. An increase in the use of plastics means that the importance of plastics has increased from the perspective of vibration transmission systems. Therefore, it is necessary to study the sound and vibration response characteristics, mode shapes, and natural frequencies of plastic composite structures [4,5,6,7].

Materials with good sound and vibration response characteristics can be applied in various fields. Materials with good response characteristics can be applied to products with important noise and vibration performance, such as laptops, TVs, air conditioners, and washing machines. Each product has an excitation source and vibration and noise from this source are transmitted to the vibration transmission system and, finally, to the product user. The application of a material with good sound vibration response characteristics in the vibration transmission system reduces the level of sound vibration transmitted to end-users, and this noise vibration reduction effect can help increase the marketability of the product. In addition, on a larger scale, materials with improved sound vibration response characteristics may be applied to automobile parts; reinforced plastic is applied to various parts, such as panels, radar housings, inverter housings, air conditioners, and heat sinks.

Some characteristics need to be considered when developing a composite material with good sound vibration response characteristics. In this study, the vibration and sound response characteristics of composite materials were measured by using the frequency response function (FRF), obtained by conducting impact tests of the specimen. The natural frequency and damping ratio of the specimens were measured by analyzing the FRF curves. To study the mechanism of the change in sound vibration response characteristics due to foaming, the dynamic characteristics and the basic mechanical properties of the material were evaluated. The basic vibration system comprises the mass, stiffness, and damping. Although there is a difference between the dynamic and static characteristics, the static modulus and dynamic stiffness tend to be proportional. The mass can also be represented by the specimen density. In a vibration system, the natural frequency is proportional to the stiffness and inversely proportional to the mass. In addition, the damping ratio tends to be inversely proportional to mass and inversely proportional to stiffness. Studies have shown that when the static modulus value decreases, the material becomes softer and, thus, the natural frequency decreases and the damping ratio increases [8]. In this study, the effects of the density, elastic modulus, damping ratio, and noise vibration response characteristics of foamed and unfoamed specimens were confirmed.

A study on the sound and vibration characteristics of fiber-reinforced plastics was conducted based on specimens manufactured by using the prepreg method or heat plate [9,10,11,12]. Unlike in previous studies, in this study, the vibration and sound characteristics of fiber-reinforced plastics for specimens manufactured through injection molding were investigated. Moreover, the focus of this study was on the difference in the dynamic characteristics between foamed specimens—formed by employing a microcellular foaming process (MCPs)—and unfoamed specimens. MCPs is a process in which a supercritical gas is dissolved in a polymer to form micropores within the micrometer unit [13]. It has several advantages, such as weight reduction, improved molding properties, sound transmission properties, reflectivity, and electrical conductivity [14,15,16,17]. Preliminary research related to this study was conducted, where the shrinkage and warpage of fiber-reinforced plastics were reduced through MCPs. Kim et al. reduced the warpage by applying MCPs and confirmed the change in fiber orientation based on the application of MCPs [18]. Ryu et al. also conducted a study on the generation of micropores and randomization of fiber orientation due to MCPs based on PA6, which is the same material used in this study [19].

In this study, an experiment was conducted using a composite containing 18% glass fiber in PA6. PA6 was selected for this study because it is a representative engineering thermoplastic resin that is both lightweight and has excellent physical properties. PA6 is tough and has excellent abrasion resistance, excellent fire resistance, fatigue limit, lubrication, shock strength, excellent strength, and rigidity. PA6 is 30% lighter than the existing PA but can maintain its physical properties. The application of PA6 is gradually expanding to improve fuel efficiency and electric vehicle AER through weight reduction in the automobile sector. The acoustic and vibration characteristics of specimens formed by injection molding process to which the MCPs was applied, which has not been addressed in previous studies, were investigated in this study. The difference in vibration response, sound response, natural frequency, and mode shape between foamed and unformed specimen was confirmed. Through a simple plate-shaped specimen, the sound vibration response characteristics based on the application of the MCPs were confirmed and the tendency was confirmed by applying the MCPs to large-sized parts with relatively complex shapes.

## 2. Materials and Methods

### 2.1. Material

The experiment was performed using glass-fiber-reinforced polymer (GFRP) reinforced with 18 wt% glass fiber in polyamide 6 (PA6). PA6 is a representative high-performance plastic, which is 30% lighter than the existing PA, although it maintains its physical properties. It has been widely used by automobile companies to reduce carbon emissions via weight reduction. The materials used in the experiments were PA6 (Kolon Plastics Inc., KOPA6 KN133HBRR, Gyeongsang, Korea). In addition, experiments were performed using inert nitrogen (N_2_) gas for microcellular foaming.

### 2.2. Microcellular-Foaming-Applied Injection Molding Process

**Figure 1:**
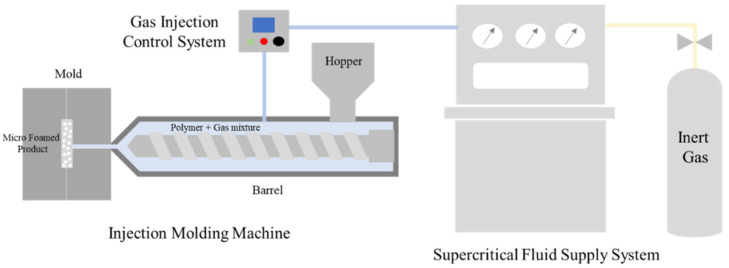
Microcellular-foaming-process applied injection molding process.

In this study, an injection molding process with MCPs was used. The MCPs melt an inert gas inside the polymer in a supercritical fluid (SCF) state and then form micropores inside the polymer through a rapid pressure drop. A schematic of the MCPs used in this study is shown in Figure 1. An injection machine with a 120 ton clamped force (Woojin Plaimm Co., Ltd., Woojin Selex-E120, Chungcheong, Korea) was used to mold the plate-shaped specimen. An injection machine with a 2300-ton clamped force (Woojin Plaimm Co., Ltd., Woojin DL2300A5, Chungcheong, Korea) was used to form the roof rack specimen. To perform the test, N_2_ gas was converted into an SCF state using a self-made supercritical fluid supply system, injected into the barrel of the injection molding machine and dissolved in the polymer. The unfoamed specimen was molded using a conventional injection molding machine. The foamed specimen, to which the MCPs was applied, foamed inside the mold by injecting N_2_ gas of supercritical fluid ecology into the barrel during injection molding.

### 2.3. Test Specimens

**Figure 2:**
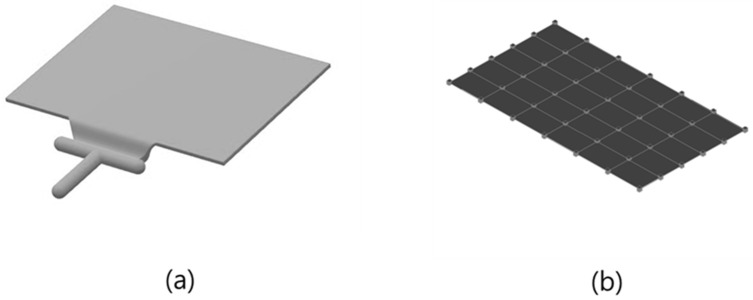
Plate specimen: (**a**) model; (**b**) geometry for mode shape.

**Figure 3:**
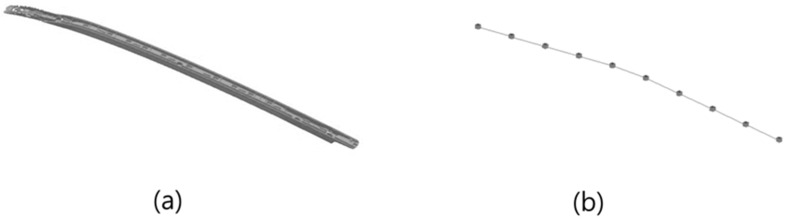
Complex product specimen: (**a**) model; (**b**) geometry for mode shape.

Two types of specimens were used in the study. The first was a plate specimen with a simple rectangular structure, as shown in Figure 2. The dimensions of the plate specimens were 150 mm × 100 mm × 2 mm; they were relatively small and had a simple, thin structure. In the case of plate specimens, the characteristics of the housing applied to small products, such as smartphones, are reflected. The second specimen was a roof-rack product as shown in Figure 3, mounted on an actual vehicle. The dimensions of the roof rack specimens were 1493 mm × 43.5 mm × 30.7 m. It was larger than the plate specimen and had complex structure and flow characteristics. Through the second specimen, we examined how the acoustic and vibration response characteristics change on a major scale when MCPs are applied to an actual large-sized product to which GFRP is applied. Because of the elongated rod shape, a change in the mode shape of the rod-shaped specimen can also be confirmed.

### 2.4. Acoustic and Vibration Characteristics

**Figure 4:**
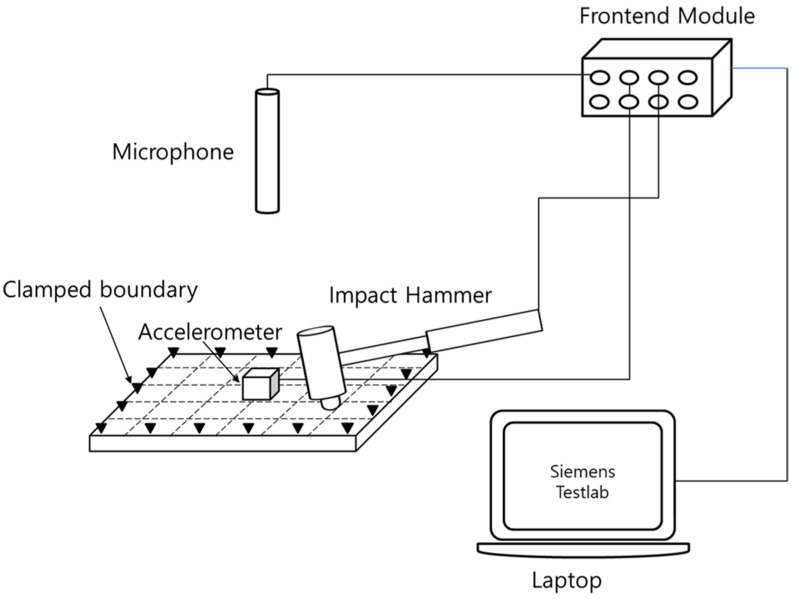
Experimental setup of acoustic and vibration characteristics.

To measure the acoustic and vibration characteristics, an experiment was conducted, as shown in Figure 4. The experiment was conducted using a frequency response function based on an impact test with an impact hammer. The equipment used was a microphone (PCB 377B02), impact hammer (PBC 086C03) accelerometer (PCB 356A15), and Frontend Module (Siemens), and the test was performed using the Siemens Testlab Software. The vibration response was measured from the excitation of the center part of the plate specimen and receiving the response of the center part. The sound response was measured using the response of the microphone at a position 100 mm from the specimen. In the case of natural frequency and mode shape, the plate specimen was tested under the boundary condition in which the four sides were clamped through a designed jig. The experiments were performed using a geometry comprising 5 × 6 30-point nodes. In the case of the roof-rack specimen, the experiment was conducted with both ends clamped by constructing a 10-point rod geometry. The FRFsum was obtained by summing the frequency response functions measured at each point. The natural frequency and mode shape were calculated using FRFsum [9,10,20].

In addition, the Q factor and the damping ratio were measured through FRF curves. The degree of damping of the vibration system was confirmed through the Q factor and the damping ratio. The Q factor is a numerical value of the sharpness of the peak in the resonant frequency domain controlled by damping in the FRF curve. The value of the Q factor is a value obtained by dividing the resonance frequency by the bandwidth at −3 dB from the peak level at the resonance frequency. The Damping ratio is a value obtained by dividing the reciprocal of the Q factor by 2. Consequently, the lower the Q factor, the higher the damping ratio, the higher the damping of the resonant frequency.

### 2.5. Mechanical Properties

In this study, the density, impact strength, tensile strength, and elastic modulus values were measured to explain the cause of the change in the noise vibration response characteristics and to confirm the mechanical characteristics of the foamed and unfoamed specimens. A density meter (MD-300S, AlfaMirage, Osaka, Japan) was used to measure the densities of the specimens. The foaming ratio, which indicates the degree of foaming of the specimen, was calculated using Equation (1). ρ_unfoamed_ represents the density of the unformed specimen and ρ_foamed_ represents the density of the foamed specimen. A universal testing machine (QMESYS Co., Ltd., QM-100T, Gyonggi, Korea) was used to measure the tensile strength and elastic modulus of the specimens. The ASTM D638 method was used as the tensile strength measurement specimen (165 mm × 19 mm × 3.3 mm). The impact strength was measured using ASTM D256. The impact strength was measured using an Izod impact tester (Salt Co., Ltd., ST-120, Incheon, Korea).

(1) $$\mathrm{Foaming}\;\mathrm{ratio}=\frac{\rho_{\mathrm{unfoamed}}-\rho_{\mathrm{foamed}}}{\rho_{\mathrm{unfoamed}}}\times 100$$

### 2.6. Cell Morphology

Scanning electron microscopy (SEM) imaging was performed to observe the formation of pores and fiber orientation in the specimen made through the MCP. The photographed specimen was taken as the cross-section of the plate specimen. Scanning electron microscopy (SEM) was used (JEOL Ltd., JEOL-7001F, Tokyo, Japan). The magnifications of the photographed images were ×200 and ×500. To confirm cell morphology, SEM images were analyzed using Image J software and the shapes of cell size and cell density were observed.

## 3. Results and Discussion

### 3.1. Vibration Response of Plate Specimen

**Figure 5:**
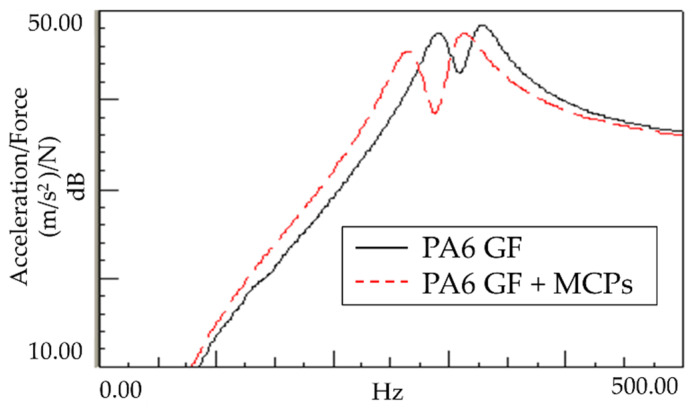
Effects of MCPs on vibration response of plate specimen.

**Figure 6:**
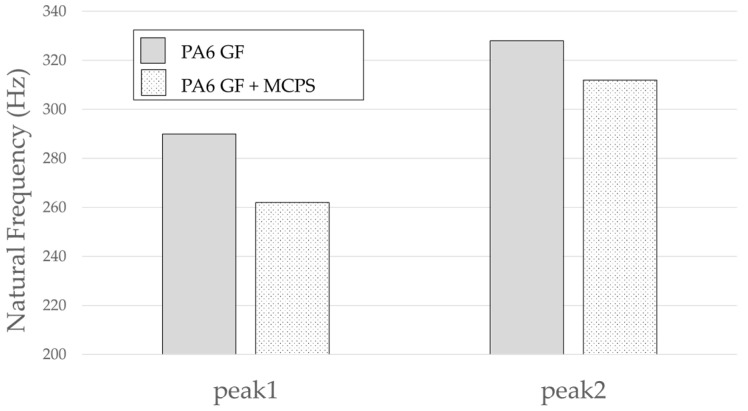
Effects of MCPs on Natural Frequency of plate specimen.

**Figure 7:**
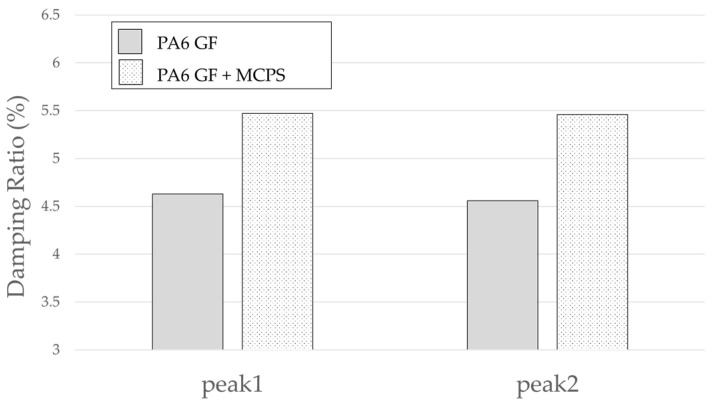
Effects of MCPs on damping ratio of plate specimen.

An experiment was conducted to determine the effect of the vibration response based on the application of MCPs. The frequency response function was obtained through the impact test of the plate specimen and the results are shown in Figure 5. In addition, the vibration characteristics in FRF curves in Figure 5 are shown in Table 1. As shown in Figure 6, the resonance frequency of the specimen made using MCPs decreases. When MCPs was applied, micropores were generated inside the specimen and the weight was reduced. However, the reduction in dynamic stiffness is greater than the mass decrease, resulting in a lower overall frequency. Another feature is that the vibration response at the resonant frequency is reduced when MCPs are applied. In addition, as shown in Figure 7, the damping ratio of the MCPs specimen increases. In general, if the amount of material decreases, the vibration response also increases because of the weakening of the dynamic stiffness, which tends to be unfavorable to vibration. However, in this case, the damping ratio increased. There are two main reasons for this result. According to the results of previous studies, when MCPs are applied, they affect the degree of crystallinity of the material, which can increase the damping ratio [21,22,23]. Another effect is the contribution from the randomized fiber orientation, which has been addressed in previous studies [16,17,24,25]. When the effect of fiber orientation on the vibration measurement is examined, the results show that the frequency and mode characteristics change according to the fiber orientation [9,10]. In addition, a study showed that when MCPs are applied to the existing GFRP, fiber orientation is relatively randomized by the increase in flowability owing to cell growth and a decrease in viscosity. Therefore, when MCPs are applied, the fiber orientation is randomized, which is expected to play a role in increasing the damping ratio of the material.

### 3.2. Sound Response of Plate Specimen

**Figure 8:**
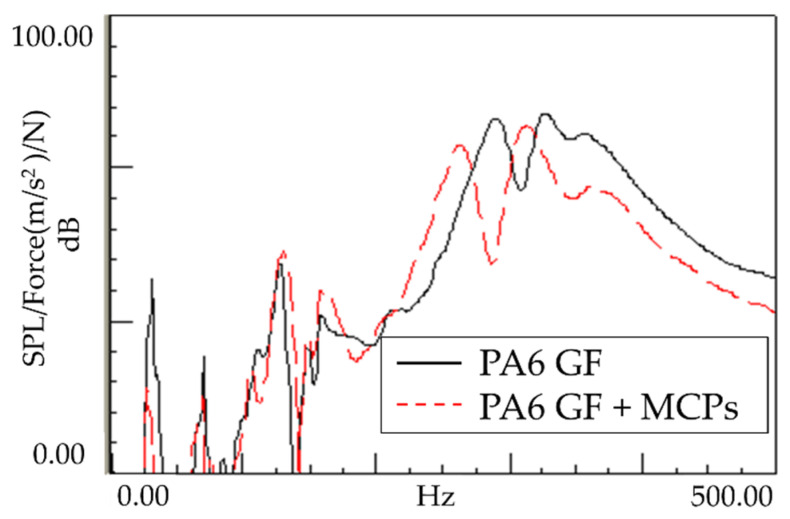
Effects of MCPs on the sound response of the plate specimen.

As shown in Figure 8, the sound response of the plate specimen was confirmed to exhibit a similar tendency to the vibration response. The resonant frequency shifted downward, and the response sensitivity decreased; this is because the vibration characteristics generated by vibrating the plate-shaped specimen were expressed as radiated sounds, exhibiting a similar tendency. This tendency appears to be advantageous for product design, whereby the product is designed to be far from the source emitting the resonant frequency. If the frequency needs to be reduced by reducing the stiffness, the sensitivity increases, and side effects can occur. In this case, the frequency can be lowered but the sensitivity is reduced accordingly. Therefore, many possible applications are expected.

### 3.3. Natural Frequencies and Mode Shapes of Plate Specimen

**Figure 9:**
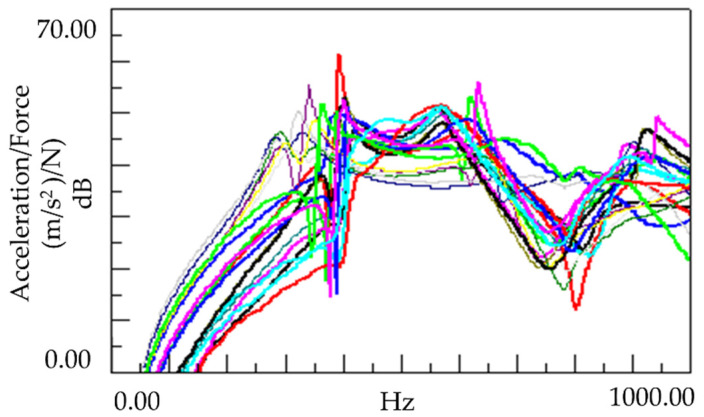
Vibration response of all points of the plate specimen.

We analyzed the sound and vibration characteristics at one point on the plate specimen, as described in Section 3.1. We divided the plate specimen into 30 points and measured the vibration response at those 30 points, as shown in Figure 9. The obtained FRFsum was utilized to determine the mode shape and natural frequency for each mode shape.

**Figure 10:**
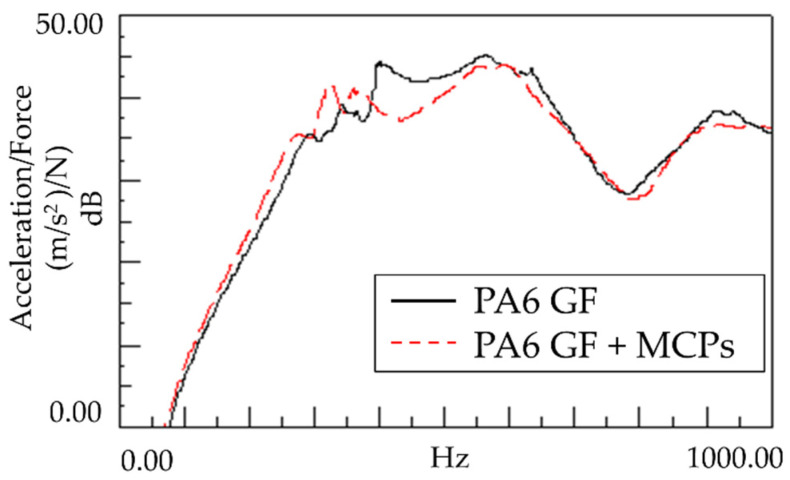
Vibration response summation of plate specimen.

**Figure 11:**
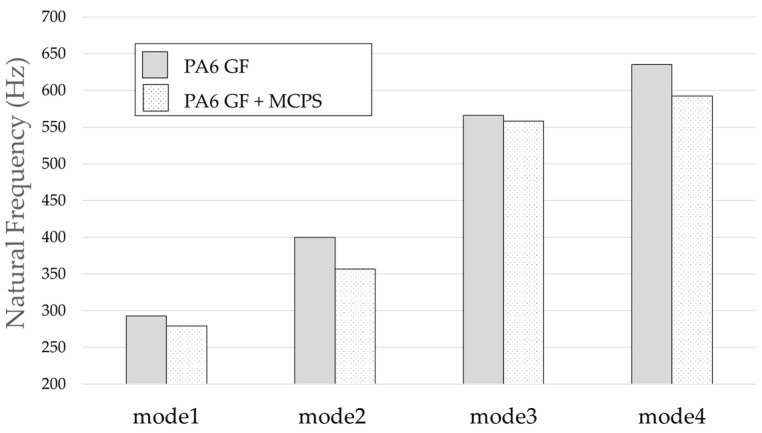
Effects of MCPs on natural frequencies of plate specimen.

**Figure 12:**
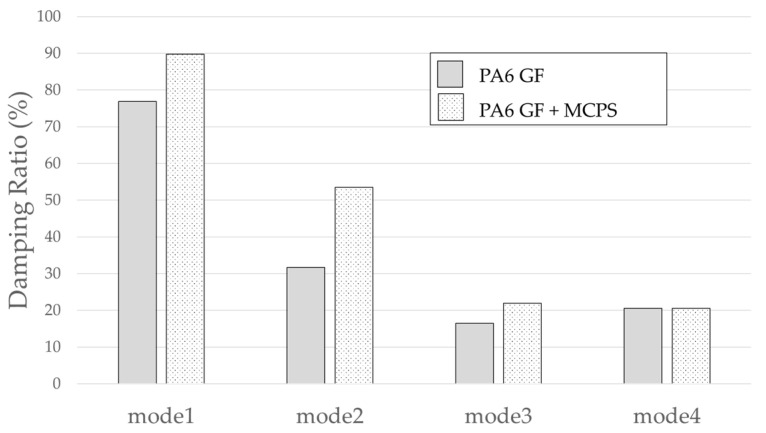
Effects of MCPs on damping ratio of plate specimen.

The result of the vibration response sum of the plate specimen are shown Table 2 and Figure 10, results were like that of the vibration response test conducted at one point. In the case of the MCPs specimen, the frequency was reduced, and the damping ratio decreased compared with the conventional PA6 GF specimen. The trends of the mode shapes obtained through the summation of the vibration responses are presented in Table 3. The natural frequency and damping ratio according to MCPs are shown in Figure 11 and Figure 12.

In the case of mode shape, it was confirmed that the unfoamed specimen and the foamed specimen showed similar shapes. However, the degree of frequency separation differed for each mode. The frequency distribution of each mode is shown in Figure 11.

The frequency of the MCPs-applied specimen was lower than that of the neat specimen. However, there was a difference in the degree of frequency shift for each mode. In the case of plate specimens, there was a difference of 23 Hz and 8 Hz in Modes 1 and 3, respectively, and Modes 2 and 4 had a difference of 43 Hz. For each mode shape, the effect of MCPs was different. In addition, the damping ratio in Mode 1 and Mode 2 increases a lot according to MCPs. However, Mode 3 has a relatively small difference, and Mode 4 has little difference. This is expected to be because the fiber orientation was randomized within the MCP, and the vibration response characteristics changed according to the shape of the mode.

### 3.4. Natural Frequencies and Mode Shapes of Roof-Rack Specimen

**Figure 13:**
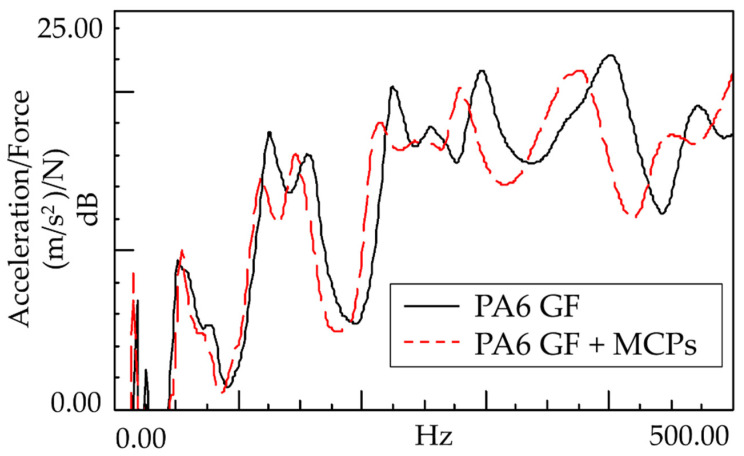
Effects of MCPs on vibration response summation of the roof-rack specimen.

In contrast to the plate specimen, the roof-rack specimen is an actual product and has a more complex structure; it is large, with a width of 1.5 m. In the case of complex large specimens through roof-rack specimens, an experiment was conducted on how the characteristics change when the MCPs is applied. In the case of the roof-rack specimen, the FRFsum was obtained by dividing the specimen by 10 points in a linear shape and summing each vibration response; the results are shown in Figure 13. Natural frequency, Q factor, and damping ratio were measured through FRF curves, which are shown in Table 4.

Even the more complex and larger roof-rack specimens exhibited similar vibrational response tendencies as the plate specimens. It can be observed that the frequency lowered and damping ratios were increased when the MCPs is applied, even in the case of large and complex specimens. The shapes of the mode shapes for each peak are presented in Table 5.

**Figure 14:**
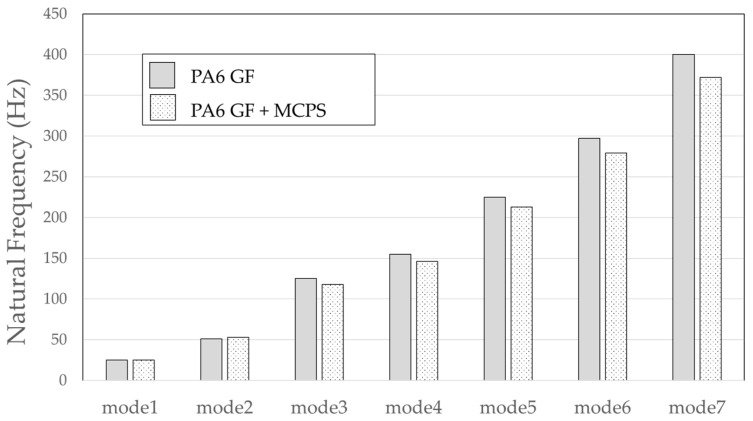
Effect of MCPs on natural frequencies of the roof-rack specimen.

**Figure 15:**
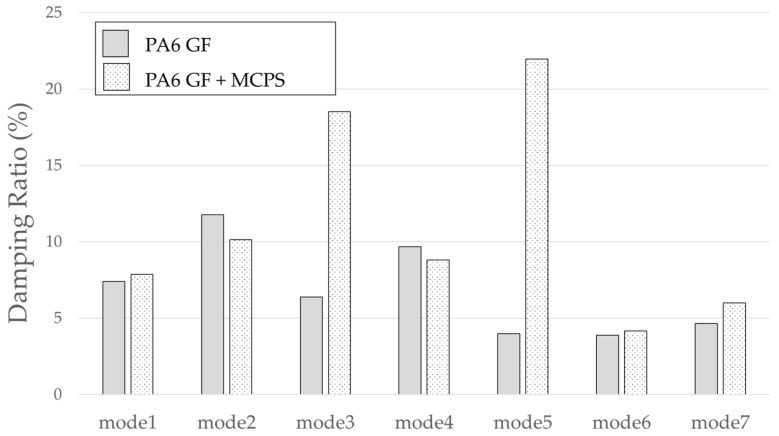
Effect of MCPs on damping ratio of the roof-rack specimen.

Because the roof-rack specimens are long and linear, mode shapes with the same shape as rods clamped at both ends can be obtained. As with the plate specimen, the frequency was lowered, and it was confirmed that the frequency response sensitivity decreased in each mode. The degree of frequency shift of each mode is shown in Figure 14. In addition, the effect of the damping ratio according to the MCPs is shown in Figure 15.

In the case of natural frequency, it can be observed that the frequency of the specimen to which the MCPs is applied is lowered and the shift level has a small difference at the beginning; then, the difference gradually occurs as it moves to the higher-order mode.

### 3.5. Mechanical Properties

**Figure 16:**
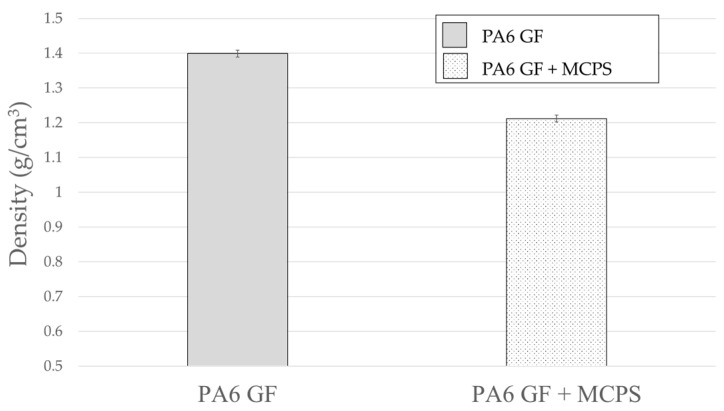
Effect of MCPs on density.

**Figure 17:**
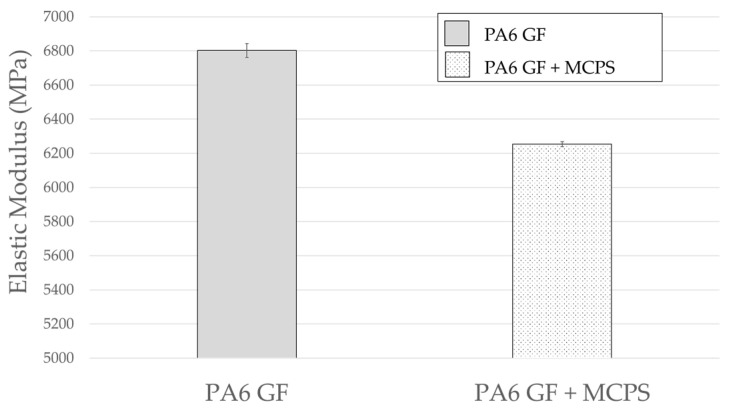
Effect of MCPs on elastic modulus.

As shown in Figure 16, the density of the MCPs-applied specimen decreases. The foaming rate is calculated to be approximately 13%. In addition, as shown in Figure 17, the elastic modulus shows a decrease in the foam specimens. A decrease in the density indicates a decrease in the mass in vibration system. When the mass decreases in the vibration system, the natural frequency increases. Conversely, the elastic modulus decreases in the same direction as the reduction in natural frequency. These impact results indicate that the natural frequency decreases. The contribution of elastic modulus reduction is greater than the effect of mass reduction. In addition, the decrease in the elastic modulus can explain the increased damping ratio. This means that the material becomes softer, and the transmitted vibration energy is converted into other energy inside the material and is lost.

**Figure 18:**
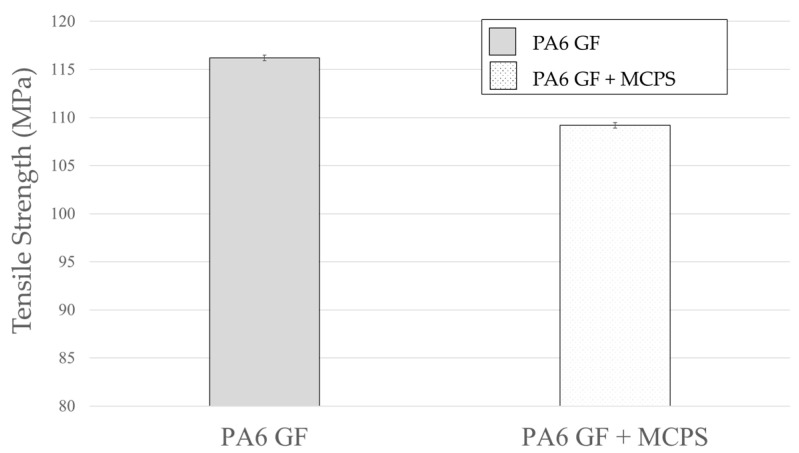
Effect of MCPs on tensile strength.

**Figure 19:**
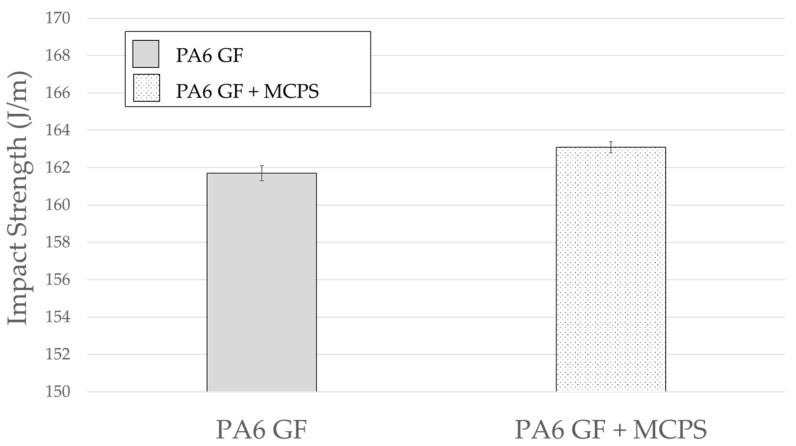
Effect of MCPs on impact strength.

The sound vibration response characteristics and the other mechanical properties of specimens foamed by using the MCPs should be verified. Therefore, in this study, the tensile strength and impact strength, which are representative mechanical properties, were measured. As shown in Figure 18, the tensile strength decreases by approximately 6%. However, it can be considered as a decline in the physical properties that can be sufficiently tolerated in preparation for a 13% weight reduction effect. In the case of the impact strength shown in Figure 19, the foamed specimen is improved; this is related to an increase in the damping ratio as the foamed specimen absorbs more energy during the impact experiment.

### 3.6. Cell Morphology

**Figure 20:**
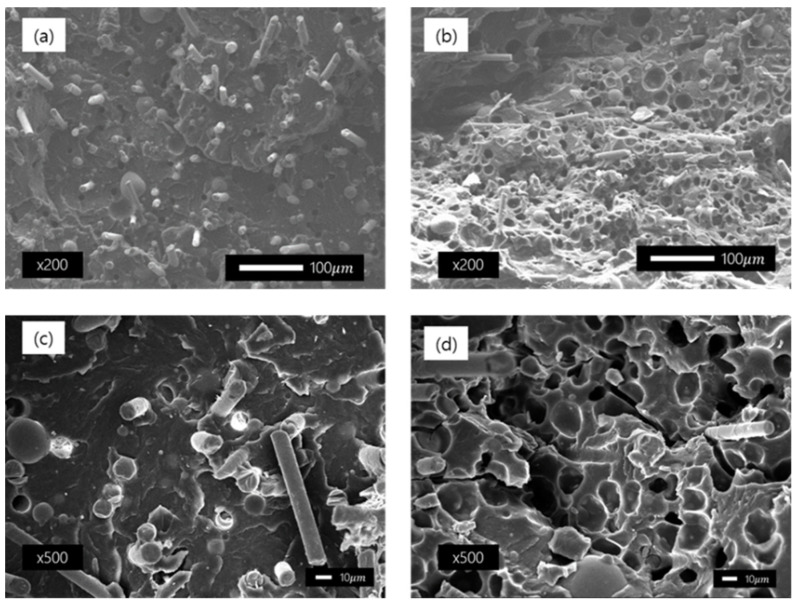
Cross-sectional images of samples taken by a scanning electron microscope: (**a**) unfoamed sample magnified 200 times; (**b**) foamed sample magnified 200 times; (**c**) unfoamed sample magnified 500 times; and (**d**) foamed sample magnified 500 times.

SEM was performed to confirm the fiber orientation and cell morphology before and after MCPs were applied to the specimens used in this study, and the images are shown in Figure 20. (a), (c) show unfoamed specimens, each measured at a magnification of ×200 and ×500. They are both cross-sections of the specimen in the flow direction, and the direction of the fibers is uniformly oriented. In contrast, the images in (b) and (d) show the results of taking a cross-section of the specimen to which MCPs are applied. To evaluate the cell morphology, the shape, size, and density of the cell were examined by using the Image J software. A closed shape cells were observed in the SEM image, with a cell size of 10–20 µm (average 17 µm) and a cell density of 8.4 × 10^7^cells/cm^3^ were formed. In addition, the fiber orientation was randomized compared to the conventional unfoamed specimen.

## 4. Conclusions

In this study, the response characteristics of sound and vibration of fiber-reinforced PA6, molded through an injection molding process to which the MCP was applied, were investigated. The sound and vibration response characteristics were measured before and after the application of MCPs to PA6 GF specimens. The results were confirmed by measuring the characteristics of a plate specimen with a simple structure and a roof-rack specimen with a relatively complex structure. The results show that in the case of the specimen foamed by applying the MCPs, the natural frequency decreased and the vibration response sensitivity tended to decrease compared with the unfoamed specimen. The mode shapes were measured to identify the tendency of the shift in each mode. The reason for the lowered natural frequency was the reduction in mass; however, the dynamic stiffness value was further reduced, lowering the frequency. The reason for the decrease in vibration sensitivity can be expected to be the effect of increasing the damping ratio owing to the increase in crystallinity when applying MCPs and the effect of the fiber orientation randomization.

Thus, the natural frequency of the vibration noise of fiber-reinforced plastics is believed to be lowered by the MCP, and the effect of reduced vibration sensitivity can be applied to various product designs in the future. When the sound and vibration characteristics of a product are tuned, the structure is advantageous as the resonant frequency of the excitation source and the part are spaced apart. Usually, if the frequency is lowered, the rigidity is reduced. However, when the MCPs are applied to fiber-reinforced plastics, as in this study, the weight and frequency decrease and the damping ratio increases. It is expected that this will be widely applied to the design of various plastic parts in the future.

## Figures and Tables

**Table 1:** Vibration characteristics from FRF curves from Figure 5.

		1st Peak	2nd Peak
PA6	Natural Frequency (Hz)	290	328
	Q factor	10.79	10.97
	Damping Ratio (%)	4.63	4.56
PA6 + MCPs	Natural Frequency (Hz)	262	312
	Q factor	9.14	9.16
	Damping Ratio (%)	5.47	5.46

**Table 2:** Vibration characteristics from FRF curves from Figure 10.

		Mode 1	Mode 2	Mode 3	Mode 4
PA6	Natural Frequency (Hz)	293	400	566	635
	Q factor	0.65	1.58	3.04	2.43
	Damping Ratio (%)	76.84	31.67	16.43	20.57
PA6+MCPs	Natural Frequency (Hz)	270	357	558	592
	Q factor	0.56	0.93	2.28	2.44
	Damping Ratio (%)	89.76	53.5	21.9	20.53

**Table 3:** Mode shapes of plate specimen.

	PA6 GF	PA6 GF + MCPs
Mode 1	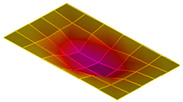	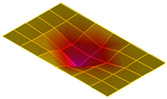
Mode 2	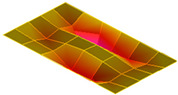	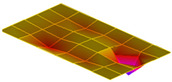
Mode 3	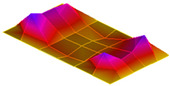	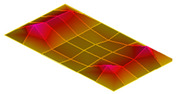
Mode 4	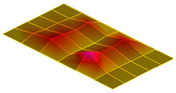	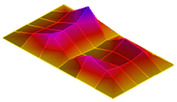

**Table 4:** Vibration characteristics from FRF curves from Figure 13.

		Mode 1	Mode 2	Mode 3	Mode 4	Mode 5	Mode 6	Mode 7
PA6 GF	Natural Frequency (Hz)	25	51	125	155	225	297	400
	Q Factor	6.76	4.24	7.82	5.17	12.56	12.89	10.72
	Damping Ratio (%)	7.40	11.78	6.39	9.67	3.98	3.88	4.66
PA6 GF + MCPs	Natural Frequency (Hz)	25	53	118	146	213	279	372
	Q Factor	6.36	4.94	2.70	5.68	2.28	12.02	8.33
	Damping Ratio (%)	7.86	10.13	18.50	8.81	21.95	4.16	6.00

**Table 5:** Mode shapes of roof rack specimen.

	PA6 GF	PA6 GF + MCPs
Mode 1	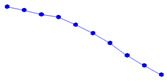	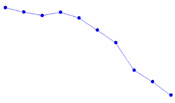
Mode 2	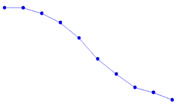	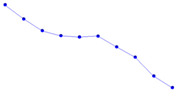
Mode 3	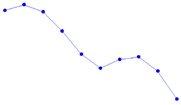	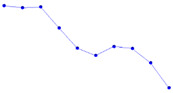
Mode 4	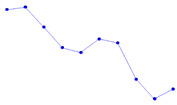	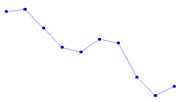

## Data Availability

Not applicable.
